# BLOOM: BLoom filter based oblivious outsourced matchings

**DOI:** 10.1186/s12920-017-0277-y

**Published:** 2017-07-26

**Authors:** Jan Henrik Ziegeldorf, Jan Pennekamp, David Hellmanns, Felix Schwinger, Ike Kunze, Martin Henze, Jens Hiller, Roman Matzutt, Klaus Wehrle

**Affiliations:** Communication and Distributed Systems (COMSYS), RWTH Aachen University, Ahornstrasse 55, Aachen, 52074 Germany

**Keywords:** Secure outsourcing, Homomorphic encryption, Bloom filters

## Abstract

**Background:**

Whole genome sequencing has become fast, accurate, and cheap, paving the way towards the large-scale collection and processing of human genome data. Unfortunately, this dawning genome era does not only promise tremendous advances in biomedical research but also causes unprecedented privacy risks for the many. Handling storage and processing of large genome datasets through cloud services greatly aggravates these concerns. Current research efforts thus investigate the use of strong cryptographic methods and protocols to implement privacy-preserving genomic computations.

**Methods:**

We propose Fhe-Bloom and Phe-Bloom, two efficient approaches for genetic disease testing using homomorphically encrypted Bloom filters. Both approaches allow the data owner to securely outsource storage and computation to an untrusted cloud. Fhe-Bloom is fully secure in the semi-honest model while Phe-Bloom slightly relaxes security guarantees in a trade-off for highly improved performance.

**Results:**

We implement and evaluate both approaches on a large dataset of up to 50 patient genomes each with up to 1000000 variations (single nucleotide polymorphisms). For both implementations, overheads scale linearly in the number of patients and variations, while Phe-Bloom is faster by at least three orders of magnitude. For example, testing disease susceptibility of 50 patients with 100000 variations requires only a total of 308.31 s (*σ*=8.73 s) with our first approach and a mere 0.07 s (*σ*=0.00 s) with the second. We additionally discuss security guarantees of both approaches and their limitations as well as possible extensions towards more complex query types, e.g., fuzzy or range queries.

**Conclusions:**

Both approaches handle practical problem sizes efficiently and are easily parallelized to scale with the elastic resources available in the cloud. The fully homomorphic scheme, Fhe-Bloom, realizes a comprehensive outsourcing to the cloud, while the partially homomorphic scheme, Phe-Bloom, trades a slight relaxation of security guarantees against performance improvements by at least three orders of magnitude.

## Background

Recent technology advances have made Whole genome sequencing (WGS) fast, accurate, and affordable. Enabled by WGS, several public initiatives [1–3] have built large cohorts of volunteers willing to share their genomes in order to accelerate biomedical research. Meanwhile, an increasing number of private players offer services related to genomic data, e.g., tracing ancestry [4]. Evidently, WGS is here to stay and the massive collection, storage, and processing of human genome data have already become a reality.

On the other side, the genome era also brings unprecedented risks for personal privacy. Genomic information uniquely identifies its owner [5] and may be misused, e.g., for surveillance [6]. The genome further carries information about an individual’s appearance, health, or predispositions [7, 8] which could cause genetic discrimination. This is aggravated by the fact that genomes remain almost stable over time and, thus, cannot be revoked or replaced once leaked or made public [9]. Since relatives share large fractions of an individual’s genome, an individual’s decision also affects the privacy of others, raising the question of kin genomic privacy [10]. Finally, the full extent of personal information that can be extracted from a person’s genome is still unknown and so are the associated privacy risks, e.g., whether it is possible to even predict human behavior from genomic analyses [11].

These significant personal privacy risks are aggravated by various attacks that have proved traditional anonymization mechanisms ineffective for genome data [12]: Wang et al. [13] re-identify individuals in a genome-wide association study (GWAS) and apply their attack to the HapMap project [3]. Sweeney et al. [14] use public demographics to re-identify a significant fraction of public profiles of the Personal Genome Project [2]. Shringarpure et al. [15] re-identify individuals in public genomic data-sharing beacons.

In response to the failure of traditional methods to protect genomic privacy, current research focuses on secure computation techniques to protect genomic privacy [16, 17]. Secure computations enable two relevant scenarios: i) *Secure collaboration:* two or multiple parties collaborate on their joint data, yet without disclosing their individual datasets. ii) *Secure outsourcing:* one or more parties outsource storage and processing of genome data to an untrusted computation cloud which remains oblivious of the data and computed analyses. In these settings, privacy-preserving variants have been proposed for, e.g., GWAS [18–20], sequence comparisons [18, 20], sequence alignments [21, 22], and genomic tests [23, 24].

The applicability of secure computation techniques is, however, limited by their significant processing, communication, and storage overheads. Scalability issues are exacerbated by the typically huge amounts of data in genomics. Secure computations and related cryptographic techniques are thus not the panacea to genomic privacy risks. Instead, their limitations and potential must be further explored and the achieved progress must be made available to non-experts. To this end, the center for integrating data for analysis, anonymization and SHaring (iDASH) [25] organizes yearly competitions to assess the state-of-the-art in secure genomic computations. The outcomes of the previous competitions are summarized in [26, 27].

In this paper, we introduce **BL**oom filter based **O**utsourced **O**blivious **M**atchings (BLOOM), our solution to the 2016 iDASH Secure Genome Analysis Competition. In Track 3 of this competition, participants were challenged to securely outsource the computations for matching a query against a database of patients’ SNPs to the cloud, e.g., to test disease susceptibility of a patient. Our key idea is to represent the query and the patient database efficiently using Bloom filters. The data owner then encrypts the Bloom filters bitwise using packing and stores the encrypted Bloom filters securely in the cloud. To enable computations on these encrypted Bloom filters, we use two different encryption schemes. In our first approach, FHE-BLOOM, we use Fully homomorphic encryption (FHE) which enables the cloud to compute a match under encryption by multiplying query and patients’ Bloom filters and to aggregate the results into single ciphertexts that are returned to the data owner. The core idea of our second approach, PHE-BLOOM, is to construct the query Bloom filter using keyed hashing which obsoletes encryption and enables efficient use of Partially homomorphic encryption (PHE). Note that this scheme slightly leaks access patterns, e.g., the cloud may learn when a query is posed twice. However, the actual contents of a query and, importantly, the patients’ data are still fully protected. PHE thus requires only a slight relaxation of the specified security requirements. We implement, evaluate, and compare both approaches on a real-world data set. FHE-BLOOM performs a disease susceptibility test on a database with 50 patients with up to 100000 variations in approximately 5 min. PHE-BLOOM notably decreases this by four orders of magnitude to 75 ms. FHE-BLOOM was ranked runner-up in the 2016 iDASH competition, while PHE-BLOOM was developed after the competition.

Before we present our two solutions, we first concisely define the problem scenario of Track 3 of the iDASH competition and briefly introduce the basic building blocks for our approaches and analyze relevant related work.

### Problem description

The iDASH Secure Genome Analysis Competition is organized yearly by the iDASH National Center for Biomedical Computing with the goal to assess and advance the state of the art of research in cryptographic techniques for the protection of genomic privacy [26, 27]. The 2016 edition of the iDASH challenge comprises three tracks [28]: addressing privacy-preserving genomic data sharing (Track 1), secure sequence comparisons in the two-party setting (Track 2), and secure matchings in the outsourcing setting (Track 3).

**Fig. 1:**
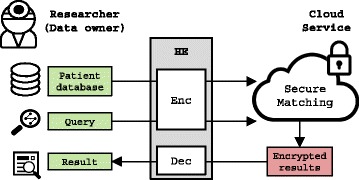
The problem scenario of Track 3 in the iDASH competition: A researcher aims to securely outsource expensive genome analysis to the cloud using homomorphic encryption

Figure 1 shows the basic scenario of Track 3 of the 2016 iDASH secure genome analysis competition [28]. We assume a researcher who owns a database containing *n* patient records. Each record comprises up to *m* SNPs given in Variant call format (VCF). The researcher also holds a query *Q* of different SNPs and wants to obtain a list of those patients that match all SNPs in the query. As the data owner is not capable of analyzing the data locally due to limited computation and storage resources, she strives to outsource computations and storage to the cloud. Due to the sensitive nature of genomic data, the cloud must remain oblivious of the computations and stored data. In this setting, the competition goal is to design a secure protocol using Homomorphic encryption (HE) that accurately and efficiently outsources computations and storage to a single cloud server. Importantly, we require a single-round protocol, i.e., after an initial setup phase the data owner poses the query and receives back the result without any intermediate interaction with the cloud server. In the following, we present the detailed requirements for solutions to this problem.

**Accuracy requirements.** The researcher aims to find exact matches, i.e., a patient matches the query *iff* all queried SNPs are contained in his patient record. The final result is a binary vector that indicates for each patient whether the query matches or not. Moreover, solutions are also judged by their ability to be generalized, e.g., to fuzzy queries or partial matches.

Performance requirements. The main optimization criterion is the query completion time which includes i) preprocessing and encryption by the researcher, ii) computations on the encrypted data in the cloud, and iii) postprocessing the final results by the researcher. Communication and processing overheads of Step iii) are tightly limited to 20 SNPs and 100 comparisons, respectively, for a database of *n*=50 patients and queries of |*Q*|≤5 SNPs [28]. Communication and storage overheads are secondary optimization goals but should still be minimized. Furthermore, overheads related to preparing and uploading the patient database should be reasonable one-time preprocessing overheads that amortize over subsequent queries.

**Security requirements.** Solutions must be secure in the semi-honest adversary model. All cryptographic primitives must offer at least 80 bits symmetric security or equivalent. The cloud must remain completely oblivious of the outsourced data and the results of the query. In particular, the length of the results must not leak the number of found matches. Furthermore, no access patterns should be leaked. The latter requirement was judged qualitatively and could be relaxed.

### Bloom filters

Both our solutions use Bloom filters [29] to efficiently represent the SNPs in queries and the patient records. Bloom filters offer a space-efficient probabilistic data structure to represent sets. They are particularly efficient when checking set membership which is a central part of our general approach.

Formally, an empty Bloom filter is a bit array *B*∈{0,1}^*l*^ of length *l* with all bits *b*_*i*_∈*B* set to zero. Before inserting elements, we fix *k* distinct hash functions $H_{1}, {\ldots }, H_{k} : \mathcal {U} \rightarrow \{0, {\ldots }, l-1\}$ that map from the universe of elements $\mathcal {U}$ into the Bloom filter *B*. To add an element $e \in \mathcal {U}$ to *B*, we compute positions *i*_1_=*H*_1_(*e*),…,*i*_*k*_=*H*_*k*_(*e*) and set the corresponding bits $b_{i_{1}}, {\ldots }, b_{i_{k}}$ to one. Similarly, to answer whether *e*∈*B*, we check whether the bits at all *k* positions *H*_1_(*e*),…,*H*_*k*_(*e*) are set. Due to hash collisions, membership checks can produce false positives, but not false negatives. The false positive probability *p* is determined by the number of hash functions *k*, the number of added elements *m*, and the length *l* of the Bloom filter. Given a fixed *m*, setting *l*=−*m* log(*p*)/ log(2)^2^ and *k*=− log(*p*)/ log(2) minimizes the false positive probability $p = \left (1 - \left (1 - \frac {1}{l} \right)^{km} \right)^{k}$.

In our second solution, we use keyed hashing when adding elements to Bloom filters, i.e., the input element to the hash functions is extended by a secret key *sk*. This prevents any party not in possession of the key *sk* to check set membership which is crucial for the security of our second approach. Keyed hashing does not affect the false positive rate and related parameters.

### Homomorphic encryption

In our approach, we encrypt the bits of Bloom filters using HE which allows performing certain arithmetic operations over ciphertexts in the encrypted domain. We utilize this property to securely match a query against the patient records under encryption such that the cloud remains completely oblivious of the processed genomic data. We distinguish three flavors of HE: i) PHE, ii) Somewhat homomorphic encryption (SWHE), and iii) FHE.

PHE schemes allow us to compute a single arithmetic operation, addition or multiplication, on ciphertexts. E.g., the Paillier [30] scheme supports additions under encryption, i.e., *E*(*x*+*y*)=*E*(*x*)*E*(*y*), while ElGamal [31] allows multiplications, i.e., *E*(*x*·*y*)=*E*(*x*)*E*(*y*). Unfortunately, these schemes do not allow the second arithmetic operation which either limits them to simple applications or requires interaction with the data owner for more complex computations.

SWHE schemes [32] allow addition and multiplication in the encrypted domain. However, homomorphic operations generate new ciphertexts with noise that accumulates over subsequent operations. Eventually, the result cannot be correctly decrypted anymore which limits the number of homomorphic operations. These schemes are thus not fully homomorphic.

FHE schemes [33–35] allow an unlimited number of additions and multiplications in the encrypted domain. Being functionally complete, they can theoretically implement any computable functionality securely by evaluating a corresponding arithmetic circuit consisting of addition and multiplication gates. Practically, the applicability of FHE schemes is limited by their significant processing and storage overheads. Especially, sequential multiplications are still very expensive such that multiplicative depth of arithmetic circuits evaluated under FHE should be minimized.

In this work, we present two solutions to securely outsource genetic disease testing, one based on the partially homomorphic Paillier scheme [30] and one based on the fully homomorphic BGV scheme [32].

### Related work

From a technical perspective, our work is related to the Private set intersection (PSI) problem which has been widely studied in the literature. We discuss the proposed solutions with respect to the special requirements in our problem setting, i.e., outsourcing to a single cloud server. The broader scope of our work is secure (outsourced) genome analysis and we conclude with a discussion of other use cases in this field. Due to the context of our work, we specifically focus our discussion of related work on *cryptographic protection* for computations over genomic data. Architectural or policy-based approaches [36, 37] are orthogonal approaches that may complement our work.

**Private set intersection.** In the standard PSI setting, client and server each hold one set of elements and they aim to compute their intersection or its cardinality securely, i.e., without either party learning the other party’s private input. PSI is an important building block for privacy-preserving protocols. Approaches range from (insecure) naive hashing [38] and semi-trusted third parties [39] over public key encryption [40, 41] to the currently most efficient protocols based on generic secure two-party computations or oblivious transfer [42]. Our approach is inspired by some of the ideas proposed in these works, i.e., the use of Bloom filters [41] and keyed hashing [38].

Most solutions to PSI are realized as two- or multi-party computation protocols, i.e., the result is computed *interactively* by two or more participants. Indeed, for some of these approaches, the majority of computations could be outsourced to two non-colluding computation peers. This has been shown for Garbled Circuits [43], Boolean sharings [21, 44], and arithmetic secret-sharing [45, 46]. Concretely, in [47], the authors outsource the set intersection protocol proposed in [42] to two untrusted computation peers. Still, these techniques do not enable outsourcing to a *single* peer and are thus inapplicable to our problem scenario. Relaxing this requirement renders these works interesting alternatives and, generally, opens up the solution space to a wide variety of existing secure computation frameworks and techniques [44, 48, 49].

In contrast, the protocols by Kerschbaum [50] and Atallah et al. [51] target outsourcing to a single server. Kerschbaum [50] proposes (outsourced) set intersection based on the Boneh-Go-Nissim encryption system [52] combined with the Sander-Young-Yung technique [53]. Atallah et al. [51] propose the secure outsourcing of linear algebra operations to a single server using secret sharing and HE. The proposed techniques could alternatively be used to implement the algorithms in our approach securely. Unfortunately, neither Kerschbaum [50] nor Atallah et al. [51] analyze the performance of their outsourcing protocols. Hence, it remains unclear whether they scale to our problem size, i.e., multiplication and addition of Bloom filters with hundreds of thousands of bits.

**Secure genome analysis.** In the related literature, many privacy-preserving variants of applications with a genomics context have been proposed. Their focus has been on GWAS [18–20, 54–56], sequence comparisons [18, 20], sequence alignments [21, 22], and statistical genomic tests [23, 24, 57]. Like ours, some of these works target the secure outsourcing setting [18, 54, 56] and make use of different flavors of HE. However, most others [19–21, 24, 55] are set in the secure collaboration setting and can only be outsourced to two or more non-colluding cloud servers. This would require a relaxation of the security requirements set forth in the iDASH 2016 challenge, as it introduces the additional security assumption that the two parties do not collude which must be further discussed in the context of genomics.

## Methods

In this section, we present our two approaches, FHE-BLOOM and PHE-BLOOM, to Track 3 of the 2016 iDASH challenge which targets secure outsourced genetic disease testing. Our core idea is to efficiently represent the SNPs in the patient database and in the query using Bloom filters and then to reduce the matching steps to operations over sets. We then design two different matching algorithms using either fully or partially homomorphic encryption. The first approach, FHE-BLOOM, facilitates a fully secure and reasonably efficient outsourcing. This solution finished runner-up in the iDASH 2016 challenge. The second approach, PHE-BLOOM, slightly relaxes security guarantees and thereby improves performance by orders of magnitude. This solution was developed after the competition and has not been ranked. In the remainder of this section, we first present a generic overview of both approaches and then explain each one in detail.

**Fig. 2:**
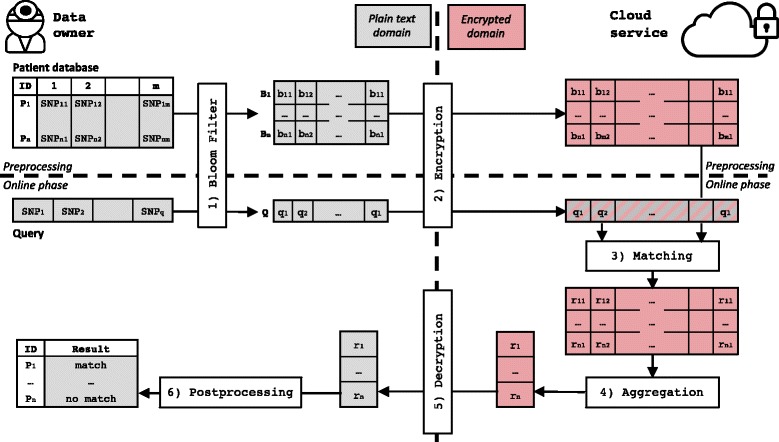
General overview of our approach: The data owner holds a database with genomes of Patients *P*_*i*=1…*n*_ which she encodes row-wise as Bloom filters (Step 1) and encrypts and uploads them to the cloud (Step 2) in the preprocessing phase. In the online phase, the data owner encodes, encrypts (only in FHE-BLOOM), and uploads her query. In the encrypted domain, the cloud matches the query to each database record (Step 3) and aggregates the results (Step 4) without ever learning the data in clear by utilizing the homomorphic properties of the chosen encryption scheme. The final results are returned to the data owner, who decrypts (Step 5) and postprocesses the results (Step 6) to obtain a list of patients that match her query

**Overview.** Figure 2 provides a combined overview of both approaches. We distinguish a *preprocessing phase* (upper part) during which the patient database is encoded, encrypted, and uploaded to the cloud and an *online phase* (lower part) that comprises all steps required to process a query. On the highest level, our goals are i) to securely outsource as much processing as possible from the data owner (left) to the cloud (right) and ii) to minimize online overheads. Both approaches proceed in the following steps: At the beginning of the preprocessing phase, the data owner holds a patient database. She creates one empty Bloom filter per patient (using keyed-hashing in PHE-BLOOM) and inserts the patient’s SNPs (Step 1). The data owner then encrypts the resulting Bloom filters bitwise before she uploads and stores them securely in the cloud (Step 2). At the beginning of the online phase, the data owner holds a fresh query to be matched against the database. In FHE-BLOOM, she transforms the query into a Bloom filter, then encrypts and uploads it to the cloud. In PHE-BLOOM, the Bloom filter is built using keyed hashing which allows to upload it to the cloud without encryption. The cloud service then matches query and patient records (Step 3) and aggregates the results in the encrypted domain (Step 4). Steps 3 and 4 are realized differently in our two approaches drawing on the different homomorphic properties of the employed HE schemes. In both approaches, the result vector contains the encrypted number of intersections between the Bloom filters encoding the query and the patient’s record. In the final step, the data owner downloads and decrypts the result vector (Step 5) and checks in a simple postprocessing step whether the number of intersections between the query and patient record is equal to the set bits in the query Bloom filter (Step 6). Note that this check finds all correct matches but may produce false matches with a probability that is upper-bounded by the configurable false positive probability of the underlying Bloom filters.

### FHE-BLOOM - genetic disease testing using FHE

The core idea of our first approach, FHE-BLOOM, is to represent patient records and queries as Bloom filters and then match and aggregate them in the encrypted domain using arithmetic operations over the Bloom filter bits. Note that this involves both multiplication (matching) and addition (aggregation) over ciphertexts which requires a fully homomorphic encryption scheme. In the following, we give the detailed steps of our first approach, FHE-BLOOM.

**Step 1: Bloom filter encoding.** In the first step, the data owner encodes the whole patient database row-wise as Bloom filters. To this end, the data owner chooses *k* hash functions *H*_1_,…,*H*_*k*_ and determines the Bloom filter length *l* according to the desired false positive probability *p* and the number *m* of SNPs that have to be inserted. Note that the choice of hash functions and the Bloom filter length remain fixed for all subsequent steps. The data owner allocates one empty Bloom filter *B*_*i*_ of length *l* for each patient *P*_*i*=1…*n*_. To insert an SNP into a Bloom filter, we first compute a unique and stable representation from the mandatory columns in VCF files to abstract from different VCF versions. We then proceed to insert *P*_*i*_’s SNPs into *B*_*i*_ by hashing each SNP *k* times and setting the corresponding bits in *B*_*i*_, i.e., $b_{i,H_{1}(\text {SNP}_{ij})},{\ldots },b_{i,H_{k}(\text {SNP}_{ij})} \forall i=1{\ldots }n, j=1{\ldots }m$.

Later, in the online phase, the data owner applies the same steps to her query to obtain the Bloom filter encoding *Q*. Additionally, the data owner counts the number of set bits cnt_*Q*_ in the Bloom filter *Q*. Note that for queries with |*Q*| SNPs, the Bloom filter *Q* will have at most cnt_*Q*_≤|*Q*|·*k* bits set.

**Step 2: Encryption and upload.** In the second step, the data owner chooses an FHE scheme, generates a key pair, and encrypts the bits in each Bloom filter. Current FHE schemes support packing techniques which offer multiple plaintext *slots* within a single ciphertext and allow operating on the encrypted plaintexts in a SIMD manner [58–60]. In the following, we denote a packed encryption of *s*_*F*_ plaintexts by . We apply packing to the Bloom filter representations of the rows of the database, i.e., we encrypt *B*_*i*_ by ⌈*l*/*s*_*F*_⌉ ciphertexts  The total *n*·⌈*l*/*s*_*F*_⌉ ciphertexts that encrypt the whole database are uploaded and stored in the cloud. In the online phase, the data owner repeats the same steps for her query *Q* and obtains ⌈*l*/*s*_*F*_⌉ ciphertexts 

**Step 3: Encrypted matching.** In the third step, the cloud filters the encrypted patient database according to the SNPs in the encrypted query. This is achieved by multiplying the encrypted Bloom filters *B*_*i*_ and *Q* component-wise, i.e.,  where ⊙ denotes the encrypted multiplication operation of the FHE scheme. Note that we slightly abuse notation here, since this step actually requires ⌈*l*/*s*_*F*_⌉ parallel ciphertext multiplications, each of which carries out *s*_*F*_ pairwise multiplications in an SIMD fashion. The resulting ciphertexts  correspond to an encrypted Bloom filter in which exactly those bits are set that correspond to the intersection of *Q* and *B*_*i*_, i.e., *r*_*i*,*j*_=*b*_*i*,*j*_·*q*_*j*_ ∀*i*=1…*n*, *j*=1…*l*.

**Step 4: Encrypted aggregation.** After Step 3, we are left with as many ciphertexts as required to store the whole patient database, i.e., a total of *n*·⌈*l*/*s*_*F*_⌉ ciphertexts. Before providing the results to the client, we aim to aggregate them further to reduce communication overheads and postprocessing. Aggregation is performed by summing up all ⌈*l*/*s*_*F*_⌉ ciphertexts used to encrypt one row into a single ciphertext. To avoid overflows, key parameters must thus be chosen such that each slot has at least log2(|*Q*|·*k*) bits.

**Step 5: Download and decryption.** After the cloud has aggregated the results, the data owner downloads and decrypts the *n* corresponding ciphertexts. The results $r_{i} = \sum _{j=1}^{l} b_{i,j} \cdot q_{j}$ hold the number of intersections between database row *i* and the query *Q*.

**Step 6: Postprocessing.** The data owner determines all *exact* matches by comparing the counts *r*_*i*_ to the number cnt_*Q*_ of set bits in the query bloom filter. If *r*_*i*_=cnt_*Q*_, then query *Q* fully matches patient *P*_*i*_ except for possibly false positives. A single false positive occurs with probability *p*. Thus, a query *Q* will produce a false match to a patient with all but one of the queried SNPs with probability *p*. Generally, if the patient matches only *i*<|*Q*| SNPs of the query, the probability of a false match decreases to *p* ^|*Q*|−*i*^.

### PHE-BLOOM - Genetic disease testing using PHE

In our second approach, PHE-BLOOM, we represent records and queries as Bloom filters as before. However, we now use a pre-image resistant keyed hash function to insert elements into Bloom filters. The query Bloom filter is then sent to the cloud without encryption since the keyed hashing already protects its contents. This enables a simpler matching algorithm as well as a higher degree of aggregation before results are sent back to the client. Note that the use of keyed hashing prevents the cloud from learning which SNPs are queried. However, the cloud learns when the same query is posed twice. While this presents a slight leakage of access patterns, performance is increased by orders of magnitude. Importantly, the patient database is still fully protected through encryption. We now explain each step of PHE-BLOOM in detail.

**Step 1: Bloom filter encoding.** Step 1 of PHE-BLOOM is similar to FHE-BLOOM with the only difference that the data owner uses keyed hashing to insert SNPs into Bloom filters. This prevents anyone who is not in possession of the hashing key from determining which SNPs are contained in a given Bloom filter.

**Step 2: Encryption and upload.** The data owner chooses a PHE scheme, generates a key pair and encrypts the bits in each Bloom filter. In contrast to our first approach, we pack Bloom filter bits *column*-wise into ciphertexts using Horner’s scheme [61]. With *s*_*P*_ slots, we obtain for the *j*th column ⌈*n*/*s*_*P*_⌉ ciphertexts  The resulting total *l*·⌈*n*/*s*_*P*_⌉ ciphertexts are uploaded to the cloud. The query is uploaded without encryption, relying on keyed hashing to protect its content.

**Step 3: Encrypted matching.** Since the cloud obtains the query bits in the clear, matching the query to the database becomes a simple matter of selecting those columns corresponding to set bits in the query, i.e., we select the encrypted column *j* *iff* *q*_*j*_=1. We thus retain at most |*Q*|·*k* encrypted columns.

**Step 4: Encrypted summation.** Before providing the results to the client, the cloud aggregates them to reduce communication and postprocessing overheads. Aggregation is done by summing up columns element-wise which is realized using encrypted additions and reduces the result’s size to only ⌈*n*/*s*_*P*_⌉ ciphertexts.

**Step 5: Download and decryption.** The data owner downloads the ⌈*n*/*s*_*P*_⌉ encrypted results from the cloud. After reception, she decrypts and unpacks them to obtain the counts $r_{i} = \sum _{j=1}^{l} b_{i,j} \cdot q_{j}~\forall i=1{\ldots }n$.

**Step 6: Postprocessing.** The occurrence of matches is decided as before by comparing the counts *r*_*i*_ to the number cnt_*Q*_ of set bits in the query Bloom filter. The probability for a false positive is exactly the same as for FHE-BLOOM.

In summary, both our approaches are based on the idea of reducing the given disease testing problem to set operations. We then identify Bloom filters as an efficient data structure that allows manipulating sets efficiently in the encrypted domain using SIMD arithmetic operations on the individual bits of the Bloom filter. Here, FHE-BLOOM is based on FHE which affords full security and competitive performance with the other solutions presented at the iDASH workshop. In comparison, PHE-BLOOM provides slightly weaker security guarantees but requires only PHE which significantly decreases overheads.

## Results

**Fig. 3:**
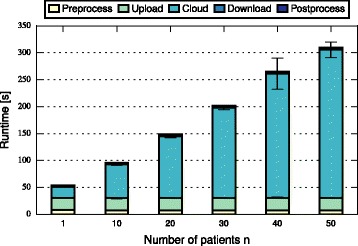
Query time of FHE-BLOOM grows linearly in the number of patients *n*

**Fig. 4:**
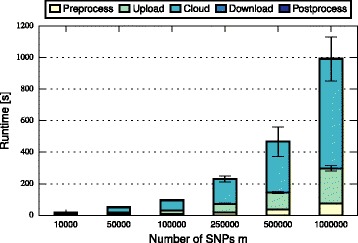
Query time of FHE-BLOOM grows linearly in the number of SNPs *m* (note the non-linear x-axis)

**Fig. 5:**
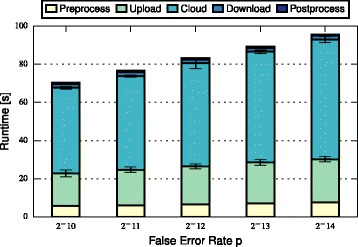
Query time of FHE-BLOOM grows linearly with exponentially decreasing *p* (note the logarithmic *x*-axis)

**Fig. 6:**
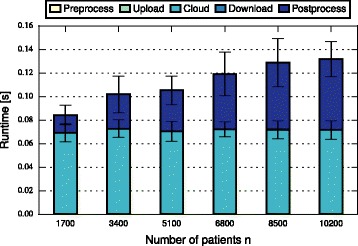
Query time of PHE-BLOOM grows linearly in ⌈*n*/*s*_*P*_⌉

**Fig. 7:**
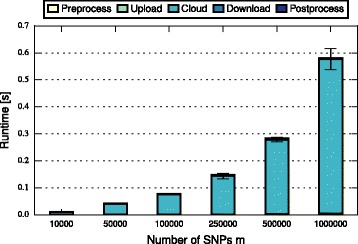
Query time of PHE-BLOOM increases linearly in the maximum number of SNPs *m* (note the non-linear *x*-axis)

**Fig. 8:**
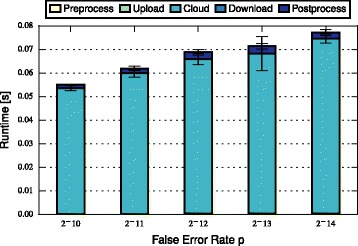
Query time of PHE-BLOOM grows linearly with exponentially decreasing *p* (note the logarithmic *x*-axis)

In this section, we discuss and compare the performance of FHE-BLOOM and PHE-BLOOM. We first formally analyze runtime and communication complexity (cf. Table 1), showing that both approaches scale linearly in the number of patients *n* and the number of SNPs *m* during setup while PHE-BLOOM has a better complexity during the query phase. We then implement both approaches to thoroughly quantify their runtime, communication, and memory overheads. First, we benchmark both approaches using the evaluation setup of the iDASH competition [28] (cf. Table 2). Afterwards, we conduct a more extensive evaluation of relevant parameters to study the performance of both approaches in greater detail (cf. Figs. 3, 4, 5, 6, 7 and 8). A qualitative discussion of the security and potential limitations is deferred to the following section.

**Table 1:** Complexity analysis of FHE-BLOOM and PHE-BLOOM: Setup overheads are similar in both approaches and grow linearly in the number of patients *n* and Bloom filter size *l* which is proportional to the number of SNPs *m*, i.e., *l*=−*m* log(*p*)/ log(2)^2^

Approach	DB setup (Client)		Query (Cloud)	Query (Client)	
	Time	Comm	Time	Time	Comm.
Fhe-Bloom	$\mathcal {O}(n \cdot l / s_{F})~\text {Enc}_{F}$	$\mathcal {O}(n \cdot l / s_{F})~\mathrm {C}_{F}$	$\mathcal {O}(n \cdot l / s_{F})~\text {Mul}_{F} + \mathcal {O}(n \cdot l / s_{F})~\text {Add}_{F}$	$\mathcal {O}(l / s_{F}) ~\text {Enc}_{F} +\mathcal {O}(n) ~ \text {Dec}_{F}$	$\mathcal {O}(l / s_{F}+n) ~\mathrm {C}_{F}$
Phe-Bloom	$\mathcal {O}(n \cdot l / s_{P})~\text {Enc}_{P}$	$\mathcal {O}(n \cdot l / s_{P})~\mathrm {C}_{P}$	$\mathcal {O}(n / s_{P}) ~\text {Add}_{P}$	$\mathcal {O}(n / s_{P}) ~\text {Dec}_{P}$	$\mathcal {O}(n / s_{P}) ~ \mathrm {C}_{P}$

**Table 2:** Competition benchmarks and test cases: i) database setup (preparing, encrypting, and uploading the database), ii) query processing in the cloud (matching query and database in the encrypted domain), iii) query overheads on the client (pre- and postprocessing the query, including overheads for up- and download), and iv) total query overheads

Setting			DB Setup (Client)			Query (Cloud)		Query (Client)		Query (Total)	
	n	m	Time	Mem.	Comm.	Time	Mem.	Time	Mem.	Time	Comm.
Fhe-Bloom											
Test 1	1	10000	5.73	91.78	26.81	3.258	86.95	7.532	91.12	10.790	27.10
Test 2	1	100000	35.24	105.77	265.44	21.075	86.81	32.938	105.65	54.013	265.74
Test 3	50	100000	1452.78	157.43	13264.54	273.922	92.98	34.385	105.53	308.307	287.71
Phe-Bloom											
Test 1	1	10000	76.77	126.06	53.29	0.008	240.40	0.001	126.06	0.009	0.03
Test 2	1	100000	752.61	128.24	533.00	0.073	2081.71	0.002	128.24	0.075	0.25
Test 3	50	100000	822.76	143.85	533.03	0.073	2081.70	0.002	143.85	0.075	0.25

Time is measured in seconds, memory and communication are measured in MBs

### Complexity analysis

We compare the runtime and communication complexity of both approaches in Table 1. Following the evaluation criteria of the iDASH competition, we distinguish the following three phases: i) *DB setup (Client)* includes all steps required for pre-processing, encryption, and upload of the patient database; ii) *Query (Cloud)* comprises all computations by the cloud over encrypted data per query; iii) *Query (Client)* includes preparation, encryption, and upload of the query as well as download, decryption, and postprocessing of the result. For simplicity, we measure runtime complexity in terms of the number of encryptions and decryptions as well as additions and multiplications in the encrypted domain. In comparison, (keyed) hashing causes only negligible overheads that are omitted in our complexity analysis. Communication complexity is measured in the number of exchanged ciphertexts.

**DB Setup:** The setup overheads for both approaches scale linearly in the number of patients *n* and length of the Bloom filter *l*. Both approaches support SIMD operations which decrease complexity by a factor 1/*s*, with *s*_*F*_ and *s*_*P*_ denoting the number of slots of the FHE and PHE schemes, respectively. The exact values of *s*_*F*_ and *s*_*P*_ depend on the chosen key, e.g., *s*_*F*_=1180 and *s*_*P*_=170 in our evaluation.

**Query (Cloud):** FHE-BLOOM requires the cloud to perform $\mathcal {O}(n \cdot l/s_{F})$ additions and multiplications. In contrast, PHE-BLOOM requires only $\mathcal {O}(n / s_{P})$ additions which is orders of magnitude more efficient.

**Query (Client):** For FHE-BLOOM, the online processing and communication overhead per query for the client are in $\mathcal {O}(l/s_F + n)$. Specifically, query preparation, encryption, and upload accounts for overheads in $\mathcal {O}(l/s_{F})$ while download and decryption account for overheads in $\mathcal {O}(n)$. PHE-BLOOM notably decreases the online overheads for the client by orders of magnitude to $\mathcal {O}(n / s_P)$. This is achieved by the use of keyed hashing which obsoletes query encryption and thereby enables much more efficient query matching and a denser packing of the results. As we will discuss in detail later on, these significant complexity improvements of PHE-BLOOM are made possible by slightly relaxing security guarantees. While a small leakage of access patterns must be tolerated, PHE-BLOOM still fully protects the patient data stored in the cloud.

### Performance evaluation

We first describe our implementations and the experimental setup, before presenting our quantitative evaluation of runtime, communication, and memory overhead of FHE-BLOOM and PHE-BLOOM. We deliberately analyze and compare only our own two approaches. A comparison of FHE-BLOOM with the competitors’ approaches has been presented by the organizers of the iDASH challenge [28]. We also emphasize that communication and memory were secondary optimization goals in the iDASH competition. Thus, we put the focus of our evaluation on the main optimization goal, i.e., the runtime per query.

**Implementation.** FHE-BLOOM is implemented in C++ based on HElib [62, 63]. All protocol steps are implemented in separate scripts that read inputs from and write outputs to disk. This allows us to process the encrypted database in chunks which becomes necessary for the larger problem sizes.

PHE-BLOOM is implemented in Python and uses our own implementation of the Paillier scheme [30, 64] and msgpack for serialization. Despite being mostly unoptimized, our implementation of PHE-BLOOM manages to keep all data in memory for the problem sizes in our evaluation. However, chunk-wise processing of the patient database as in FHE-BLOOM is also straightforward to implement for this approach if memory consumption needs to be reduced. Both implementations use the pybloom implementation of Bloom filters which uses SHA512 as hash function. For keyed hashing, we append a secret key to the hashed input just as done for salted hashes. Both implementations are available online to facilitate reproducibility of our results [65].

**Experimental setup.** We perform experiments on a desktop client (Ubuntu 14.04 LTS, 8 CPUs at 3.40 GHz, 8 GB RAM) and a server (Ubuntu 14.04 LTS, 16 CPUs at 2.60 GHz, 32 GB RAM) that communicate over a Gigabit LAN. As in the competition, we limit execution on both machines to 4 cores. All cryptographic primitives are configured to offer at least 80 bit symmetric security.

We use the dataset provided by the organizers of the iDASH competition and vary the parameters that determine the performance of our approach, i.e., number of patients *n*, maximum number of SNPs per patient *m*, and false positive probability *p*. If not stated otherwise, we fix *n*=10,*m*=100000, and *p*=2^−14^. The setup overheads are measured over 5 independent runs and online overheads over 30 independent runs. We report the mean and the standard deviation.

Since the provided dataset contains no queries, we use random matching and non-matching queries in our evaluation. However, we emphasize that for fixed *n*, *m*, and *p* the performance of our approach does not in any way depend on which or how many SNPs are queried and whether they match or not.

#### Runtime

Table 2 compares how both approaches perform in the three test cases of the iDASH competition. We observe that overheads for preparing, encrypting, and uploading the database are within the same order of magnitude for both approaches. Overheads of FHE-BLOOM are lower for small *n* but grow quickly, while PHE-BLOOM has a higher overhead first but, interestingly, does not increase when moving from *n*=1 in Test 1 and 2 to *n*=50 in Test 3. This is due to the different packing strategy of PHE-BLOOM that packs column-wise with *s*_*P*_=170 slots which is easily big enough to fit each column into a single ciphertext. To draw a fair comparison between both approaches, we measure the asymptotic runtime per patient for fixed *m*=100000. FHE-BLOOM then requires 27.98 s (*σ*=2.39 s) per patient in the database, while PHE-BLOOM only requires 5.40 s (*σ*=0.12 s). While these overheads are significant, they are still reasonable and are computed only once. We now focus on the main optimization goal, i.e., the online runtime per query.

For FHE-BLOOM, the query runtime increases linearly in *n* and *m* and is in the order of seconds for Test 1 and 2 and in the order of minutes for Test 3. In contrast, the online overheads of PHE-BLOOM are smaller by three orders of magnitude. Figures 3, 4, 5, 6, 7 and 8 further break down query turnaround time for different *n*, *m*, and *p*. The plots show mean and standard deviation for i) query preparation on the client, ii) query upload, iii) processing the encrypted data in the cloud, iv) results download, and v) postprocessing the results.

For FHE-BLOOM, we observe linear growth in the number of patients *n* (Fig. 3) as well as in the number of SNPs *m* (Fig. 4), while runtime increases only logarithmically with decreasing false positive probability *p* (Fig. 5). We observe that in all cases, cloud overhead dominates the query turnaround time. The overheads for the client are much smaller in comparison, the postprocessing overheads being barely noticeable. This is a desirable property of our system as it fulfills the main goal of outsourcing, i.e., to minimize client overheads.

For PHE-BLOOM, we measure the same query turnaround time for all *n*<*s*_*P*_=170, i.e., all previous choices. This is expected since overheads of PHE-BLOOM increase only stepwise every *s*_*P*_-many patients as up to *s*_*P*_ patients are packed into a single ciphertext. To confirm this behavior, we evaluate PHE-BLOOM for numbers of patients that are multiples of *s*_*P*_, i.e., *n*=10·*s*_*P*_,20·*s*_*P*_,…,60·*s*_*P*_ (Fig. 6). Indeed, we observe a linear growth of the postprocessing overheads due to the increased number of decryptions. Interestingly, the fixed costs of iterating over all bits in the query dominate the cloud overheads such that the overhead for adding columns, which grows in *n*, is barely perceivable. As expected, preprocessing and upload overheads remain constant since they do not depend on *n*. Notably, PHE-BLOOM scales easily to thousands of patients. As for FHE-BLOOM, the online runtime of PHE-BLOOM grows linear in *m* (Fig. 7) and logarithmic in *p* (Fig. 8). Our complexity analysis (Table 1) does not capture this behavior since the measured overheads (Figs. 6, 7 and 8) are due to iterating the query Bloom filter, i.e., plaintext operations that are not considered in our complexity analysis.

In summary, the runtimes of FHE-BLOOM are within the order of minutes even for the largest parameter choices which we deem reasonable for practical deployments. In comparison, PHE-BLOOM is at least three orders of magnitude faster and answers queries on a database with thousands of patients in milliseconds.

#### Communication

Table 2 shows the communication overheads for i) the upload of the encrypted patient database and ii) the upload of the query plus the download of the results. FHE-BLOOM has lower overheads for a small number of patients *n* which is due to the previous observation that PHE-BLOOM’s different packing strategy pays off only for larger numbers of patients. To confirm this behavior, we measure the asymptotic overheads per patient in the database. For *m*=100000, FHE-BLOOM asymptotically uploads 265.30 MB (*σ*=0.01 MB) per patient during setup of the database and a fixed 265.29 MB (*σ*=0.00 MB) per query irrespective of *n*, while downloading only 0.45 MB (*σ*=0.00 MB) per patient in the database. In contrast, PHE-BLOOM asymptotically only uploads 3.13 MB (*σ*=0.00 MB) per patient during setup and 0.25 MB (*σ*=0.00 MB) per query while needing to download only 1.58 B (*σ*=0.00 B) per patient. Thus, communication overheads in PHE-BLOOM are two orders of magnitude smaller than in FHE-BLOOM for large patient databases.

#### Memory

Table 2 shows cloud’s and client’s memory overhead during the setup and query phase. In the following, we discuss memory consumption in the setup and query phase by the example of Test 3, i.e., *m*=100000 and *n*=50. For both approaches, the client’s memory consumption in both phases is fairly low with a maximum consumption of 156.86 MB (*σ*=1.78 MB) and 143.85 MB (*σ*=0.38 MB) in FHE-BLOOM and PHE-BLOOM, respectively. Clearly, these overheads are manageable even by constrained clients. For FHE-BLOOM, the cloud’s memory consumption is fairly low as well and amounts to only 92.97 MB (*σ*=0.15 MB). This is achieved by storing the encrypted database on disk and reading and processing it in chunks of the desired size. While this introduces perceivable I/O overhead, it becomes necessary for the larger problem settings, e.g., in Test 3 where the encrypted database has a storage size of 13264.54 MB (*σ*=0.01 MB) and is too big to be kept in the memory. In contrast, the encrypted database in our second approach, PHE-BLOOM, is at least one order of magnitude smaller and can be kept in memory entirely. While this saves I/O overhead, it increases memory consumption to 2081.70 MB (*σ*=0.03 MB). In scenarios where the cloud has constrained memory, the database could also be processed chunk-wise to reduce memory overhead, similarly to our implementation of FHE-BLOOM. Overall, the cloud’s memory overheads are still feasible in both approaches even for large problem sizes.

## Discussion

In this section, we discuss our two approaches, FHE-BLOOM and PHE-BLOOM, w.r.t. their security guarantees and their limitations and potential extensions.

### Security discussion

We briefly discuss security of our approaches in the semi-honest adversary model. In the semi-honest model, all parties correctly follow the protocol but may try to infer additional information from the protocol transcript. The semi-honest model has many applications and advantages: It allows for efficient protocols and protects against insider and outsider attacks in settings where both parties are not actively cheating. In our setting, the cloud does not contribute any private data. Thus, we only have to show that the cloud learns nothing from the protocol transcript about the patient database, the query, and the result.

In FHE-BLOOM, the client begins by uploading the encrypted database to the cloud. While the cloud learns the number of patients *n* in the database, we argue that it learns nothing else about the content of the database due to the semantic security of the employed encryption scheme. In particular, semantic security guarantees that the cloud cannot learn any partial information from the ciphertexts, e.g., whether two patients have the same SNPs. Since all encrypted Bloom filters have the same size, the cloud does not even learn the individual number of SNPs per patient but only the configured maximum number *m*. In the second step, the client poses multiple queries to the cloud. Again due to the semantic security of the encryption scheme, the cloud learns nothing about the queried SNPs and cannot even distinguish whether the same query was posed twice. Thus, no access patterns are leaked. Following, the cloud computes the matching and aggregation steps. All operations in the cloud are performed on encrypted data, hence any intermediate results remain encrypted. Importantly, the matching and aggregation steps always access the complete database and all bits in the query such that data access patterns are completely independent of the database, the query, and the result. We thus argue that the processing steps reveal nothing to the cloud and even withstand timing side-channel attacks. Finally, the cloud returns the encrypted results to the client. The results always consist of *n* ciphertexts independent of the number of matches found, and hence do not leak information to the cloud. We thus conclude that FHE-BLOOM is fully secure in the semi-honest model and even withstands timing attacks.

In PHE-BLOOM, the client begins with the upload of the encrypted database as before. The employed Paillier scheme is semantically secure and thus the previous security arguments apply. Different to FHE-BLOOM, the query is not encrypted in PHE-BLOOM. Instead, we use a pre-image resistant keyed hash function to map SNPs into the query Bloom filter, where only the client knows the hashing key. The use of a secret hashing key prevents the cloud from mounting a brute-force attack to learn which SNPs are queried. Note that we use the key only to salt the hash and do not require, e.g., robustness against length-extension attacks as provided by keyed hash functions for message authentication such as HMAC [66]. However, keyed hashing is deterministic and thus not semantically secure. In consequence, the cloud can distinguish whether queries are different or the same and if they overlap. This presents a slight leakage of access patterns which we argue might be tolerable in scenarios where high performance is paramount. The same but no additional access patterns are leaked during matching, where the cloud selects the database columns corresponding to the bits set in the query Bloom filter. Aggregation is then performed completely under semantically secure encryption without any further information leakage. The returned results and their length are, as before, independent of the found matches. We thus conclude that, besides slightly leaking information about the posed queries, PHE-BLOOM is secure in the semi-honest model.

PHE-BLOOM shows that tolerating a slight leakage of access patterns leads to significant performance speed-ups. To conclude the security discussion, we briefly point out a different relaxation of the security requirements that can achieve performance improvements. As we have noted in our analysis of related work, a significant part of the research on secure genomic analysis relates to the secure collaboration setting. In this setting, state-of-the-art techniques such as Yao’s Garbled Circuits [67], the Goldreich-Micali-Wigderson protocol [68], or secret-sharing-based SMC [69], have often found to be more efficient than (fully) homomorphic encryption. Unfortunately, they are interactive protocols that require collaboration of multiple parties. These techniques are thus inapplicable in a *strict* secure outsourcing setting. Relaxing the strict requirement of outsourcing to a single party, however, renders these established secure multiparty computation techniques applicable in our problem scenario. Instead of outsourcing to a single cloud provider, the data owner would securely split data across two cloud services and instruct them to carry out the genomic computations together using any of the established secure computation techniques. It is important to note that this relaxation introduces the additional security assumption that the two parties do not collude which must be further discussed in the context of genomics.

### Limitations and extensions

Our evaluation has shown that the most expensive step is the setup of the patient database in both approaches. Although these overheads amortize over multiple queries, we aim for a system that does not require repetition of the whole setup phase each time the database has to be changed or query functionality is extended. We thus first analyze the costs for modifying the encrypted and outsourced patient database at a later point in time, i.e., adding, deleting, and modifying patient records. Afterwards, we briefly discuss the flexibility of our two approaches in answering different types of queries beyond exact matches.

**Modifying the encrypted database.** In FHE-BLOOM, it does not matter whether patient records are uploaded in one batch during setup or whether they are added later on. In both cases, we need to construct one Bloom filter per patient, encrypt, and upload it. The runtime overheads are thus exactly the asymptotic costs per patient during database setup reported earlier. To delete a patient the cloud simply deletes the corresponding ciphertexts. A record can be modified by simply replacing it. In comparison, single row-wise operations, such as adding, deleting, and modifying a patient, are more expensive in PHE-BLOOM. Each of these operations requires the client to create and upload exactly *l* new encryptions. This is due to the different packing strategy which packs the data of *s*_*P*_ patients column-wise into *l* ciphertexts. However, this allows us to operate on a batch of *s*_*P*_ consecutive rows in an SIMD fashion. Thus, operations should be performed on batches of *s*_*P*_ patients whenever possible such that overheads amortize. Also, PHE-BLOOM is more efficient for column-wise operations, e.g., adding, deleting, or modifying particular SNPs of the patients.

**Answering further query types.** Both FHE-BLOOM and PHE-BLOOM are designed to compute exact matches. However, the results can also be interpreted as the size of the intersection between query and patient records to probabilistically estimate the degree of a *partial* match. In particular, this allows answering *negative* queries efficiently, e.g., which patients do *not* show certain variations. Note that both approaches allow us to compute arbitrary linear combinations of the patients’ SNPs. This allows us to answer *weighted* queries, e.g., enabling disease tests where certain SNPs are more critical than others. Concretely, this can be implemented in both approaches by assigning integer weights instead of bits to the Bloom filter slots and interpreting the final results as the weights of the matchings. Finally, with Bloom filters at the core of both approaches, a wide variety of Bloom filter extensions might apply to add support for, e.g., range queries [70] or locality-sensitive hashing for fuzzy queries [71].

## Conclusions

The grave privacy risks and failure of traditional protection schemes make evident the need for strongest possible protection for genomic data. Currently, best protection is achieved by secure computation protocols that share data only in cryptographically protected form such that the data is never learned by third parties in clear. The 2016 Secure Genome Analysis Competition, organized by the iDASH center, aims to assess the state of the art of these techniques and to make them available to non-experts. In this paper, we presented two solutions to Track 3 of this competition, i.e., secure outsourced disease testing. Both of our solutions are based on Bloom filters but differ in the use of homomorphic encryption to realize computations over encrypted data in the cloud. Our first approach, FHE-BLOOM, uses fully homomorphic encryption to protect the patient database and the posed queries while fully outsourcing storage and computations. In our second approach, PHE-BLOOM, we slightly relax security guarantees by allowing a slight leakage of access patterns. This enables efficient use of partially homomorphic encryption which significantly improves performance while still realizing a comprehensive outsourcing and full protection of patient data. Concretely, FHE-BLOOM queries a database of 50 patients with up to 10000 disease markers in 308.31 s (*σ*=8.73 s), while PHE-BLOOM performs the same test case over three orders of magnitude faster, in only 0.07 s (*σ*=0.00 s). Both approaches support flexible and efficient management of the outsourced data, e.g., adding or modifying patient records, and may be extended to support further query types beyond exact matches that were required in the competition. FHE-BLOOM was ranked runner-up in the iDASH competition, while PHE-BLOOM was developed only afterwards but presents an exciting alternative when high performance is of paramount importance.

## Abbreviations

FHE: Fully homomorphic encryption
GWAS: Genome-wide association study
HE: Homomorphic encryption
iDASH: Integrating data for analysis, anonymization and SHaring
PHE: Partially homomorphic encryption
PSI: Private set intersection
SIMD: Single instruction multiple data
SNP: Single nucleotide polymorphism
SWHE: Somewhat homomorphic encryption
VCF: Variant call format
WGS: Whole genome sequencing
